# Movement of *Striacosta albicosta* (Smith) (Lepidoptera: Noctuidae) Larvae on Transgenic *Bt* and Non-*Bt* Maize

**DOI:** 10.3390/insects14060524

**Published:** 2023-06-05

**Authors:** Débora G. Montezano, Thomas E. Hunt, Priscila M. Colombo da Luz, Kelsey Karnik, Stephen D. Kachman, Ana M. Vélez, Julie A. Peterson

**Affiliations:** 1 Department of Entomology, University of Nebraska-Lincoln, Lincoln, NE 68503, USA; 2 Haskell Agricultural Laboratory, Department of Entomology, University of Nebraska-Lincoln, Concord, NE 68728, USA; 3 West Central Research, Extension & Education Center, Department of Entomology, University of Nebraska-Lincoln, North Platte, NE 69101, USA; 4 Department of Statistics, University of Nebraska-Lincoln, Lincoln, NE 68588, USA

**Keywords:** larval movement, insect resistance, *Bt* maize crops, insect behavior

## Abstract

**Simple Summary:**

The planting of genetically modified crops that express insecticidal proteins is central to modern pest management, particularly for maize and cotton in the United States. An understanding of how the presence of transgenic proteins can affect the movement and feeding behavior of insect pests will allow us to better design resistance management strategies to prolong the use of this technology. In this study, the western bean cutworm, a regionally important pest of maize, was studied in artificial arena and on-plant experiments. Two more artificial experiments did not indicate significant changes to young caterpillar behavior when exposed to maize tissues expressing transgenic proteins; however, the more field-realistic and longer exposure period studies did indicate that young caterpillars would abandon plants that expressed transgenic proteins more often that those that did not. This finding has particular importance for the use of integrated versus structured refuges.

**Abstract:**

Exposure of lepidopteran pests to *Bacillus thuringiensis* (*Bt*) proteins has been shown to affect the behavior of larvae, including increased movement and avoidance of *Bt*-expressing plants or diet. Therefore, we hypothesized that the behavior of western bean cutworm, *Striacosta albicosta* (Smith) (Lepidoptera: Noctuidae), an important pest of maize, could be affected when exposed to *Bt* plants. To test this hypothesis, we conducted a series of artificial arena and on-plant experiments to determine *S. albicosta* neonate behavior when exposed to *Bt* and non-*Bt* plant tissue. Video tracking experiments presented neonate larvae with the choice of *Bt* or non-*Bt* pollen in a Petri dish for 15 min while being video recorded for analysis with EthoVision software. This study showed an increase in mean velocity and total time spent moving for larvae in the presence of Cry1F vs. non-*Bt* when compared with Vip3A vs. non-*Bt* or Cry1F vs. Vip3A. However, there was no difference in total distance moved or time spent in the food zone for all scenarios. Maize tissue choice experiments allowed neonatal larvae the choice of feeding on *Bt* or non-*Bt* tassel or leaves for 9 h in Petri dish arenas. This experiment showed that larvae preferred tassel tissue over leaves but did not indicate that larvae could distinguish between *Bt* and non-*Bt* tissue. In contrast, on-plant experiments (including a whole plant neonate dispersal study under controlled conditions and an in-field silking behavior experiment) indicated that the presence of Cry1F and Vip3A *Bt* toxins increased plant abandonment, suggesting that larvae are able to detect and avoid *Bt* toxins. The discrepancy of these results is likely due to the on-plant studies providing more field-realistic environmental conditions and a longer duration of exposure to Bt toxins for the behavioral experiments. Our results represent the first steps in understanding the complex behavior of *S. albicosta* when exposed to *Bt* plants. A better understanding of the response of larvae when exposed to *Bt* traits can aid in the management of this pest, particularly for the design of resistance management strategies and refuge design.

## 1. Introduction

Genetically modified maize, *Zea mays* L., containing transgenes from the bacterium *Bacillus thuringiensis* (*Bt*), has been grown in the United States since 1996. *Bt* maize was first developed for primary lepidopteran pests, such as European corn borer *Ostrinia nubilalis* (Hübner) and southwestern corn borer *Diatraea grandiosella* (Dyar), for which the toxin levels expressed in transgenic plants are considered high-dose [1]. However, pest pressures in maize have changed over time due to various ecological and agronomic factors, including the range expansion of species different from the original targets [2]. Consequently, lepidopteran species formerly considered secondary pests, such as the western bean cutworm *Striacosta albicosta* (Smith), are now being managed with *Bt* transgenic plants [3,4]. Importantly, *Bt* toxin expression in transgenic maize is not considered high-dose for some of these pests, including western bean cutworm *S. albicosta* [5,6,7,8,9]. 

*S. albicosta* is an important pest of maize and dry beans (*Phaseolus vulgaris* L.) that has expanded its range since 1999 from the western Great Plains of the United States eastward into the Great Lakes region of the U.S. and Canada [10]. Ear feeding causes significant yield loss due to kernel damage and secondary fungal infections [4,10,11,12]. In maize, *S. albicosta* oviposit on the upper surfaces of leaves, with a preference for plants immediately before tassel emergence [11,13,14]. Egg mass size ranges from 2 to 345 eggs per mass, with an average of 85 eggs per mass [15]. Newly hatched larvae initially consume the chorion, and then move to the tassel within the whorl to feed on tassel tissue and developing pollen. When larvae reach the third to fourth instar, they will move to the ear where they feed until reaching the sixth to seventh instar, then move to the soil to overwinter as prepupae [12,16]. 

In addition to within-plant movement, larvae of many Lepidoptera species exhibit significant plant-to-plant movement (e.g., [17]). Pannuti et al. [18] observed *S. albicosta* to typically move down and across maize rows within a ~1.7 m radius around an infested plant, with the maximum distance moved of 6.8 m. Hagen [11] reported a 1.8–3.0 m diameter movement zone around maize plants infested with *S. albicosta* egg masses. Some Noctuidae pests can disperse even further distances during the larval stage. First, instar larvae can use silk to suspend themselves from plant surfaces and balloon through the air to adjacent plants or other objects by the wind [17,19]. This ballooning behavior has been reported for 31 species of Lepidoptera [19] but remains poorly studied in *S. albicosta*.

The most common methods for *S. albicosta* management are the planting of *Bt* maize and/or insecticide application after reaching the economic threshold, as determined by in-field scouting [3,4,10,20]. Currently, there are only two *Bt* proteins expressed in commercially available maize that have had efficacy against *S. albicosta*: Cry1F and Vip3A [14,21,22]. The Cry1F toxin provided approximately 80% efficacy in protecting maize from *S. albicosta* when it was first released [14]. However, resistance has developed to Cry1F among *S. albicosta* populations in the United States [9] and Canada [6]. Baseline bioassay data show that *S. albicosta* neonates are highly susceptible to Vip3A [7]. However, the estimated Vip3A protein exposure for later instar larvae is less than the LC_99_ [23]. 

In an effort to delay resistance to *Bt* toxins, the U.S. Environmental Protection Agency (EPA) requires the planting of non-*Bt* refuge to maintain a source of insects carrying *Bt* susceptible alleles [24]. The refuge allows the survival of susceptible individuals, reducing the probability that resistant insects will mate with each other, consequently reducing the frequency of resistant individuals. Important assumptions of the refuge strategy are that resistance alleles are rare, recessive, and not sex-linked [25,26]. Depending on product and region, non-*Bt* refuges may be planted in a block comprising 5, 20, or 50% of the land (also called “structured refuge”), or, since 2010, as a seed blend, sometimes called “integrated refuge”, “blended refuge”, or “seed blends”, where 5–10% of the seed planted is non-*Bt*, with the remaining 90–95% expressing *Bt* toxins. Integrated refuges are often attractive to growers and trait developers (compared to structured refuge) due to their ease of deployment during planting and the assurance that refuge compliance will be met. However, despite the increasingly common practice of planting blended refuges, this scenario can cause concerns for resistance management of lepidopteran pests that feed on the ears of maize plants. Sublethal exposure to *Bt* toxins can be exacerbated when larvae move between *Bt* and non-*Bt* plants, or in instances when feeding occurs on cross-pollinated kernels. Cross-pollination between *Bt* and non-*Bt* plants can result in refuge ears that do not effectively function as a refuge, but instead >90% of kernels may contain one or more *Bt* proteins in a mosaic of expression levels [27,28]. Larval movement amongst adjacent plants in seed blends can also decrease effective *Bt* toxin exposure and increase the rate at which resistance evolves by creating a functionally low-dose environment that facilitates survival of heterozygous resistant larvae and decreases survival of susceptible larvae [29]. Another detrimental component of larval movement entails the movement of susceptible larvae away from non-*Bt* and onto adjacent *Bt* plants, which serves to reduce the effective refuge size [30]. Additionally, increased survival of late instar heterozygotes, which complete the first part of their larval stage on non-*Bt* plants, then move to *Bt* plants when they are older, larger, and less susceptible to *Bt* toxins, could increase the rate of resistance development [23]. If target pests are able to detect *Bt* toxins in plants, they may move away from plants or tissues expressing *Bt* toxin(s) and consequently be exposed to a lower *Bt* toxin concentration, increasing the probability of heterozygote(s) survival and potentially accelerating the evolution of resistance [26,29,31]. 

The presence of toxins has been demonstrated to alter the behavior of insects on their host plant [32]. Behavioral studies show that the exposure to *Bt* toxins in maize increases the likelihood of insects moving between plants [33,34]. Goldstein et al. [35] observed *O. nubilalis* neonates to more frequently abandon *Bt* maize plants via silking than non-*Bt* maize. Davis and Coleman [36] observed that, when exposed to transgenic maize expressing Cry1Ab protein, *O. nubilalis* neonates were rarely on *Bt* maize tissue when compared to non-*Bt* maize tissue, and they exhibited an increase in wandering behavior, declining consumption rates over time, and a decrease in total leaf consumption. Such behaviors may expose insects to a sub-lethal dose of *Bt* toxins, fostering the development of resistance [26,29,31].

The long-term use of *Bt* toxins depends on the use of tactics to delay or manage the development of resistance. Some of the critical factors to consider when developing an IRM plan are associated with pest biology and behavior (e.g., movement of larvae and adults), and in which scenarios a pest will more likely develop resistance (e.g., toxin avoidance) [37]. Although a few studies have examined *S. albicosta* larval movement [12,18] and interactions with other Lepidoptera [38], none have investigated *S. albicosta* larval behavior when exposed to *Bt* plants. Therefore, research is needed to elucidate the behavior of *S. albicosta* larvae when they encounter *Bt* maize. The objective of this research was to determine the differences in *S. albicosta* neonate dispersal behavior when exposed to *Bt* (Vip3A or Cry1F) and non-*Bt* maize in Petri dish arenas using visual observation and video tracking software, and under increasingly realistic conditions of whole plants in controlled environmental conditions and in the field. Maize plants expressing two different *Bt* proteins were used in this study to describe scenarios where the pest population tested is both susceptible (Vip3A) and resistant (Cry1F) to the transgenic traits [3,9]. 

## 2. Materials and Methods

### 2.1. Source of Striacosta albicosta and Maize Plants

All experiments were conducted using egg masses laid by field-collected adults. Moths were collected from light traps located near Grand Island, NE, USA (40°54′43.10″ N, 98°16′33.60″ W) [16]. Each morning, moths were transferred to the laboratory and placed into rearing cages (63.5 × 63.5 × 63.5 cm) (Bug Dorm, MegaView Science Co., Ltd., Talchung, Taiwan) containing late vegetative stage pinto bean plants for oviposition. Adult diet consisted of a 5% sucrose and 0.2% ascorbic acid solution provided by a 150 mm × 15 mm sponge placed in a Petri dish (4.7 cm in diameter × 0.7 cm height, Pall Corporation, Port Washington, NY, USA). Eggs were collected daily from the pinto bean plants and held in a growth chamber at 26.6 ± 1 °C, 70–80% relative humidity (RH), and 16:8 h (L:D) photoperiod, and monitored daily for hatching [39]. 

Maize expressing Cry1F (SmartStax^®^, DKC 61-55 RIB, DeKalb; it also expresses Cry1A.105, Cry2Ab2, Cry3Bb1, and Cry34/35Ab1 proteins that do not affect *S. albicosta* [14,21,22]), Vip3A (Agrisure Viptera, G99z33-311A, Syngenta; it also expresses Cry1Ab that does not affect *S. albicosta* [14,21,22]), or no *Bt* proteins (DKC 61-52, DeKalb, from the same hybrid family as the Cry1F plants; non-*Bt* maize from the same hybrid family as the Vip3A-expressing maize was not available for this research) were grown under identical field conditions and standard agronomic practices for the region, with no insecticide applications, at the University of Nebraska-Lincoln’s West Central Research and Extension Center in North Platte, NE, USA (41°05′23.6″ N 100°46′20.4″ W). Cry1F and Vip3A expression were confirmed using ImmunoStrip^®^ for *Bt*-Cry1F (Agdia, Inc., Elkhart, IN, USA, STX 10900/0050) and ImmunoStrip^®^ for Vip3A (Agdia, Inc. STX 83500/0050). 

### 2.2. Video-Tracking

The video-tracking experiment was performed to determine the effect of pollen tissue on larval movement and preference in artificial arenas after a short duration of exposure to *Bt*. This experiment was conducted in Petri dishes in the laboratory. Each 60 mm diameter × 15 mm height Petri dish (Fisher Scientific, Hampton, NH, USA) was divided into two halves with a 0.2 mg pile of pollen (defined as the “food zone”) placed at the center of each half (located 2 cm apart from each other). Pollen was collected by removing the entire tassel from a maize plant and shaking over white paper. Treatments consisted of: (1) Vip3A and non-*Bt* pollen; (2) Cry1F and non-*Bt* pollen; or (3) Cry1F and Vip3A pollen. One 48 h-old *S. albicosta* first instar larva (allowed to feed since hatching on artificial diet with no *Bt* toxins [39]) was transferred to the center of each Petri dish with a paintbrush at the start of the experiment. Each Petri dish was recorded with a Dino-Lite AD413T-12V camera (Big C, Torrance, CA, USA) for 15 min at approximately 24.4 °C, 50% RH. Each larva participated only once in a scenario. Each treatment was replicated 30 times, totaling 19 h of observation. Automated video-tracking software (Ethovision^®^ XT 14 [40]) was used to evaluate the mean velocity (mm/s), total distance moved (cm), time (s) larvae spent in the food zone, and the total time (s) spent moving or not moving. 

Velocity, percentage of time spent moving, and distance moved were analyzed with a one-way analysis of variance (ANOVA) using the PROC GLIMMIX procedure in SAS. Since the time spent in and out of the food zone were not normally distributed variables, these data were analyzed using non-parametric Kruskal-Wallis tests (one-way ANOVA on ranks) in SAS to evaluate the differences between groups. 

### 2.3. Maize Tissue Choice

**Figure 1:**
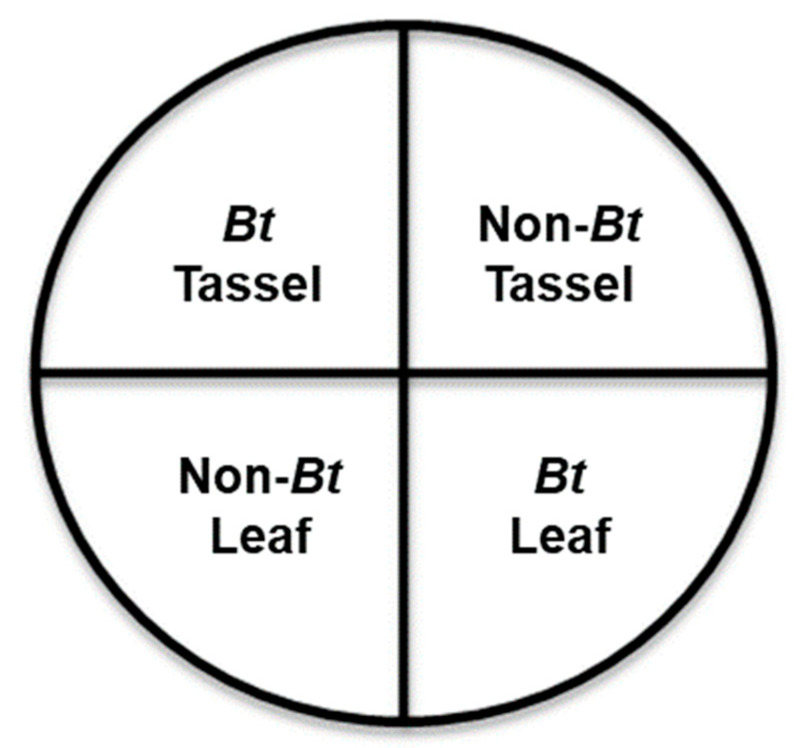
Experimental design for the maize tissue choice experiment conducted in a 60 × 15 mm Petri dish divided into four quadrants. *Bt* tassel and leaves expressed either Cry1F or Vip3A proteins.

The maize tissue choice experiment was conducted to determine whether larvae were able to choose between *Bt* and non-*Bt* plant tissue within a 9 h exposure period in an artificial arena. Experiments were performed in 60 mm diameter × 15 mm height Petri dishes (Fisher Scientific) divided into four equal quadrants with a piece of plant tissue placed at the center of each quadrant with 1.5 cm distance between tissues (Figure 1). Plant tissue was obtained from tassel stage maize and collected immediately prior to the start of each trial to assure freshness of the tissue. Experimental treatments consisted of: (1) one-piece Vip3A tassel, one-piece non-*Bt* tassel, one-piece Vip3A leaf tissue, and one-piece non-*Bt* leaf tissue; or, alternatively, they consisted of (2) one-piece Cry1F tassel, one-piece non-*Bt* tassel, one-piece Cry1F leaf tissue, and one-piece non-*Bt* leaf tissue. Tassel tissue pieces were 0.5 mg each, and leaf tissue pieces were 1.7 cm diameter leaf discs cut using a number 13 cork borer (Humboldt H-9663 Plated Brass Cork Borer Sets with Handles). To prevent maize tissue from desiccation, 15 mL of a 10 g mL^−1^ agar solution was dispensed into each Petri dish and allowed to cool prior to the experiment. The agar was scored using a small spatula, and the leaf discs and tassel tissue were positioned vertically in the agar. The relative position of the plant tissue was randomized for each arena. One 48 h old first instar *S. albicosta* larva was transferred to the center of each Petri dish with a paintbrush at the start of the experiment. Larvae were randomly selected from different egg masses to minimize effects of genetic similarity. The position of the larva was recorded by visual observation every 30 min for nine consecutive hours (total of 18 observations per larva). The position of the larva was recorded as one of five locations: (1) *Bt* Tassel, (2) *Bt* Leaf, (3) Non-*Bt* Tassel, (4) Non-*Bt* Leaf, or (5) Off-tissue. Each treatment was replicated a total of 72 times (24 replicates per day for three consecutive days). All experiments were conducted at approximately 24.4 °C, 50% RH, and 16:8 h (L:D) photoperiod. 

Statistical analyses were performed separately for comparisons of Vip3A vs. non-*Bt* and Cry1F vs. non-*Bt* using a generalized linear mixed model, where the proportion of larvae at each location was fit to a binomial distribution with a random effect of Petri dish [41,42] using the PROC GLIMMIX procedure in SAS (SAS software SAS, v. 9.4, SAS Institute Inc., Cary, NC, USA). Overall significance for location was evaluated first (*p* < 0.05) before comparing group means with approximate pairwise *t*-tests.

### 2.4. On-Plant Neonate Dispersal

The on-plant neonate dispersal experiment was conducted to determine whether neonate larvae would abandon *Bt*-expressing plants more often than plants that did not express *Bt* over a period of 24 h under controlled environmental conditions. Cry1F, Vip3A, and non-*Bt* maize plants, grown in the field as described above, were transferred to 4-L plastic pots when they reached tasseling stage. Individual maize plants were placed inside PVC pipe frame cages (1 × 1 × 2 m) and sealed with plastic film (Best Choice Plastic Wrap, Kansas City, KS, USA). The bottom of the cage was covered with a water dish to serve as a trap to collect any neonates dropping vertically from the plant. Unbaited yellow sticky cards (Trécé Inc., Chelsea, OK, USA) were placed in a ring along the inside surface of the cage walls to serve as traps to collect horizontally ballooning neonates. The experimental unit consisted of one plant (Cry1F, Vip3A, or non-*Bt*) inside one of these cages. Each trial consisted of two replicates of each of the three treatments. The arrangement of the cages was randomly assigned for each trial. Trials were repeated six times, resulting in 12 replicates for each treatment. To initiate a trial, approximately equal-sized purple egg masses (indicating less than 24 h from hatching) were selected, the number of eggs in each mass was recorded, and one egg mass per plant was placed onto the top leaf of each maize plant to mimic *S. albicosta* oviposition behavior [12]. Plants were destructively sampled 24 h after the egg mass hatched. All unhatched eggs and neonates that could be found were recorded. Recovered neonates were assigned to four categories based on the location where they were observed: (1) Tassel, (2) Upper Leaves, (3) Lower Leaves, (4) Off-plant, and (5) Silks. Upper Leaves were those located above the ear, and Lower Leaves were those located at or below the ear. Neonates on leaves, tassels, and silks were considered to have stayed on the plant, and those on the sticky traps or in the water pan were considered to have abandoned the plant (Off-plant). All experiments were conducted at approximately 24.4 °C, 50% RH, and 16:8 h (L:D) photoperiod.

The proportions of neonates found at each location (Tassel, Upper Leaves, Lower Leaves, Silk, and Off-plant) were analyzed with a generalized linear mixed model with a binomial distribution [41,42] using the PROC GLIMMIX procedure in SAS (SAS software v. 9.4, SAS Institute Inc.). A 3 × 5 factorial treatment structure was used to estimate the influence of the three independent variable treatments (Cry1F, Vip3A, or non-*Bt* plants) on the proportion of larvae found at each location, with replicate plants considered a random effect. Overall significance for the main effects and interaction of treatment and location were evaluated first (*p* < 0.05), and corresponding means of interest were compared using approximate pairwise *t*-tests. 

### 2.5. Silking Behavior

The silking behavior experiment was conducted to determine whether, after a 24 h pre-exposure period, larvae showed plant-abandoning behavior by silking off from *Bt* expressing plants more often than non-*Bt* plants in the field. This experiment was performed under field conditions at the University of Nebraska-Lincoln’s West Central Research and Extension Center in North Platte, Nebraska, USA (41°05′23.6″ N 100°46′20.4″ W). Treatments consisted of Cry1F, Vip3A, and non-*Bt* plants planted in adjacent rows (seeds were planted 3–4 cm deep and 10–12 cm apart in 80–85 cm rows). Previous experiments with *O. nubilalis* have shown that larvae do not exhibit a difference in silking behavior if only briefly exposed to *Bt* plants [35]. Using plastic containers and fresh *Bt* plants, we pre-exposed neonates (1 h after hatching) for 24 h to each treatment before evaluating silking behavior. After the pre-exposure period, one neonate was randomly chosen using a camel’s hair paintbrush (size 2) attached to the neonate’s silk strand and placed on the top leaf of a tassel stage plant. Thus, all neonates tested in this experiment had previously silked at least once. After placing the larva on the plant, observations were made for 15 min. Each neonate was observed for incidence of silking, distance moved (if a larva moved less than 10 cm it was not considered for this experiment), and if larvae fed or not. Five replications were made every day at 9 am CDT (all three treatments were conducted simultaneously) over the course of ten consecutive days, resulting in a total of 50 replications per treatment. During the experimental period, the average temperature was 26.4 °C (range = 17.8–39.4 °C), and the average wind velocity, measured by using an airflow indicator (Kestrel 1000 Pocket Wind Speed Meter Anemometer, Thomastown, Australia), was 25.55 cm/s (range = 10–46 cm/s). Data were analyzed using Fisher’s Exact test with the PROC FREQ procedure in SAS (SAS software v. 9.4, SAS Institute Inc.). 

## 3. Results

### 3.1. Video-Tracking

Analysis of *S. albicosta* first instar larvae exposed to pollen and evaluated with the video tracking software EthoVision XT 14 showed that there was no evidence of an overall significant difference in velocity between treatments (F = 2.30; df = 2, 85; *p* = 0.1068; Table 1). A significant difference among treatments was found for the percentage of time spent moving (F = 6.64; df = 2, 85; *p* = 0.021). Larvae spent significantly less of their time moving when exposed to Vip3A vs. Non-*Bt* pollen when compared to Cry1F vs. Non-*Bt* (*p* = 0.0016) and Cry1F vs. Vip3A (*p* = 0.023) pollen. There was no evidence of differences among treatments for the total distance moved (F = 1.79; df = 2, 85; *p* = 0.1733), time spent out of the food zone (*χ*^2^ = 2.2321; df = 2; *p* = 0.3276), and time spent in the food zone (*χ*^2^ = 0.2020, df = 1, *p* = 0.6531; Vip3A vs. Non-*Bt* (*χ*^2^ = 0.6472, df = 1, *p* = 0.4211), Cry1F vs. Non-*Bt* (*χ*^2^ = 0.0862, df = 1, *p* = 0.7691), and Vip3A vs. Cry1F (*χ*^2^ = 2.1135, df = 2, *p* = 0.3476) (Table 1). 

### 3.2. Maize Tissue Choice

**Figure 2:**
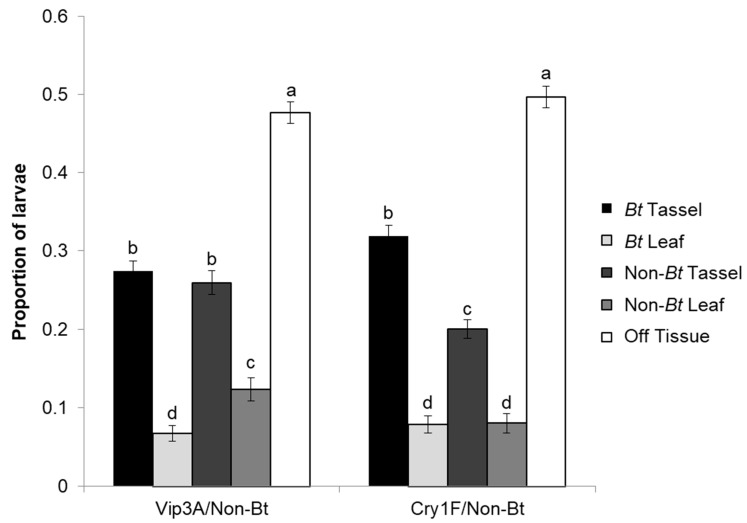
Proportion (mean ± SEM) of *Striacosta albicosta* first instar larvae observed within a 9 h period on *Bt* and non-*Bt* plant tissues (tassels or leaves) or off-tissue within maize tissue choice experiment treatments for Cry1F and Vip3A (*n* = 144 Observations = 2592). Bars with different letters indicate significant differences among locations within treatment (*p* < 0.05).

Results from the maize tissue choice experiment showed that the Vip3A vs. non-*Bt* treatment had significantly different proportions of first instar larvae observed on *Bt* leaf tissues, non-*Bt* leaf tissues, and off-tissue (F = 95.03; df = 4177; *p* < 0.0001; Figure 2). The majority of the larvae were observed off-tissue, differing significantly from all the other locations (*p* < 0.001), but there was no significant difference between observations on *Bt* tassel and non-*Bt* tassel (*p* = 0.4441). The non-*Bt* leaf location was also significantly different from all other locations (*p* < 0.0018), and *Bt* leaf was the location with the lowest proportion of observations compared to all other locations (*p* < 0.001) (Figure 2). Results from the analysis of the Cry1F vs. non-*Bt* treatment showed that there was a significant difference in the proportion of larvae observed among *Bt* leaf tissues, non-*Bt* leaf tissues, and off-tissue (F = 118.99; df = 4, 177; *p* < 0.0001). The highest proportion of observations was made off-tissue, differing significantly from all other locations (*p* < 0.001), followed by *Bt* tassel (*p* < 0.001) and non-*Bt* tassel. The lowest proportions of observations were on *Bt* leaf and non-*Bt* leaf, with no difference between them (*p* = 0.9120) (Figure 2). 

### 3.3. On-Plant Neonate Dispersal

Overall, the majority of neonates (56.20%) were recovered off-plant, followed by on the upper leaves (29.05%), tassels (7.53%), silks (3.77%), and lower leaves (3.45%). The factorial analysis showed a significant effect of treatment (F = 9.98; df = 2, 132; *p* < 0.0001), location where larvae were recovered (F = 511.50; df = 4132; *p* < 0.0001), and an interaction between treatment and location (F = 11.78; df = 8, 132; *p* < 0.0001). Given the significant interaction between treatment and location, differences for treatments within individual locations were evaluated.

**Figure 3:**
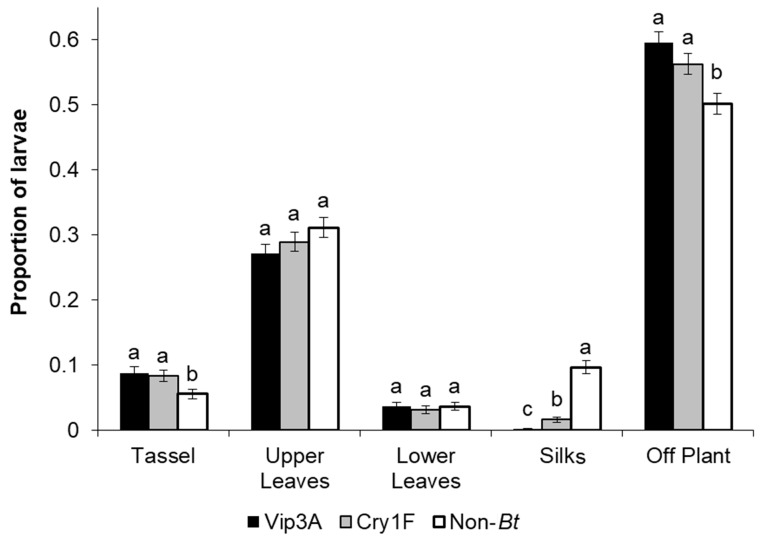
Proportion (mean ± SEM) of *Striacosta albicosta* neonates recovered at tassels, upper leaves, lower leaves, silks and off-plant 24 h after hatching for Vip3A, Cry1F, and non-*Bt* treatments in the on-plant neonate dispersal experiment. Comparisons between treatments were made by pairwise *t*-tests. Bars with different letters indicate significant differences between treatments within a location (*p* < 0.05).

A higher proportion of larvae were found at the tassels on Vip3A (*p* = 0.0070) and Cry1F (*p* = 0.0179) plants compared with non-*Bt* plants (with *p*-values indicating significant differences), although there was no evidence of differences between the two types of *Bt* plants (*p* = 0.7303) (Figure 3). No differences between treatments were found for the proportion of larvae recovered at upper leaves and lower leaves. There were significant differences between all three treatments for larvae recovered at the silks (Figure 3). Non-*Bt* plants had the highest proportion of larvae on silk when compared to Cry1F (*p* < 0.0001), and Vip3A (*p* < 0.0001) and Cry1F also had a higher number of larvae than Vip3A (*p* = 0.0089) on the silks (Figure 3). There was a higher proportion of larvae recovered off-plant in the Vip3A (*p* < 0.0001) and Cry1F (*p* = 0.0008) treatments compared with non-*Bt*, although there were no differences between the two types of *Bt* plants (*p* = 0.3153) (Figure 3). 

### 3.4. Silking Behavior

**Figure 4:**
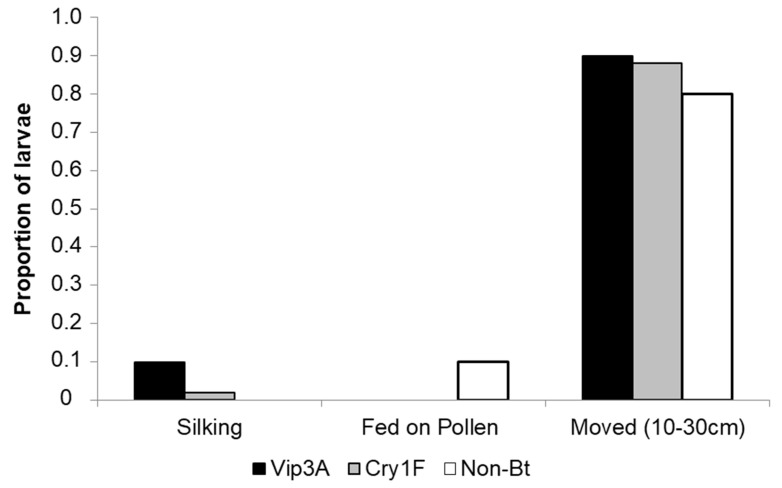
Proportion of *Striacosta albicosta* neonates that silked, fed on pollen, or moved between 10 and 30 cm when exposed to Vip3A, Cry1F and non-*Bt* plants. Larvae were pre-exposed for 24 h to Vip3A, Cry1F, or non-*Bt* maize tissue prior to being placed on plants in the field with matching expression and observed for 15 min.

A Fisher’s Exact test showed a marginally significant relationship between treatment and the proportion of larvae that demonstrated silking behavior (*p* = 0.0478). Silking behavior was observed for 10% (*n* = 5 of 50 evaluated) of the neonates exposed to Vip3A, 2% (*n* = 1 of 50 evaluated) of the neonates exposed to Cry 1F, and 0% (*n* = 0 of 50 evaluated) of the neonates exposed to non-*Bt* plants (Figure 4). Feeding behavior on pollen was observed in a higher proportion of larvae in the non-*Bt* treatment (10%; *n* = 5 of 50 evaluated) compared to the Cry1F and Vip3A treatments (0% for both; *p* = 0.0107) (Figure 4). The majority of the neonates moved between 10 and 30 cm during the observation time (96%, 90% and 76% for Vip3A, Cry1F, and non-*Bt*, respectively), with no evidence of a significant relationship among treatments (*p* = 1.000; Figure 4). 

## 4. Discussion

*S. albicosta* is most frequently reported to demonstrate a sequential feeding pattern in the field, with the earliest instars feeding on tassel tissue before moving down the plant to the ears [12]. Following this behavior, we expected to recover the highest proportion of larvae that did not abandon the plant on tassels in the on-plant movement study. However, the highest proportion of on-plant larvae were found on the upper leaves. Our observations were taken 24 h after hatching, which should have been sufficient time for neonates to travel from the egg masses to the tassel tissue, based on field observations that neonates will hatch, feed on the egg chorion, and then disperse from the egg mass within 1–12 h from hatching (Melotto et al., in prep). Therefore, our results may have been affected by the phenology of the maize plants: pollen shed had just begun at the time of the experiment, with all tassels completely emerged and visible, causing pollen shed on upper leaves, providing food, and altering the upward movement of the larvae. While the sequential movement from egg mass to tassel to ear is most typical, it is important to note that if crop growth stage is further along, particularly if tassels are more mature, neonate larvae may reduce the time spent feeding on tassels and travel towards the ear sooner [12]. Results from the maize tissue choice experiments (conducted in Petri dishes) more closely followed the expectation that tassel tissue would be preferred to leaves. *S. albicosta* first instar larvae were observed in highest proportions off-tissue, followed by on tassel tissue, then on leaf tissue. Compared to the whole plant study, the tassels used in the maize tissue choice experiment were less developed and had not begun desiccation. These results confirm the preference of first instar larvae for fresh tassel tissue before pollen has shed and become available on leaves.

Results from video tracking trials demonstrated that first instar larvae did not change velocity, move greater distances, or spend significantly more time outside of the food zone when exposed to *Bt* toxins; these results do not support the hypothesis that larvae exposed to *Bt* toxins will increase wandering and avoid *Bt* toxins, as shown for other Noctuidae species [26,43,44]. This may be explained by the short duration of larval exposure to the maize pollen (15 min) in our study. Significant differences were only observed for the percentage of time spent moving, with significantly less time spent moving when larvae were exposed to Vip3A vs. non-*Bt*, which could be explained by the toxicity of Vip3A pollen causing a reduction in movement after larvae were exposed to it. *Bt* protein expression can vary based on numerous factors, including transgenic event, plant tissue, and environmental factors. For the transgenic event TC1507 (expressing Cry1F proteins), mean protein expression was highest from pollen (27 ng/mg tissue dry weight), followed by leaf (20), stalk (8.8), root (5.7), and grain (3.7) [45,46]. A similar pattern of expression can be found for the transgenic event MIR162 (expressing Vip3A proteins), although mean protein expression was highest for leaf (107.7 ng/mg tissue dry weight), followed by silk (97.4), pollen (47.1), pith (31.7), and root (28.3) [47].

In the tissue choice experiment, no significant differences were observed between the proportion of larvae found on Vip3A *Bt* and non-*Bt* tassel, as well as between *Bt* and non-*Bt* leaves for both proteins. It has been reported that larvae of other lepidopteran pests, such as European corn borer *O. nubilalis*, eastern spruce budworm *Choristoneura fumiferana* (Clemens), and lightbrown apple moth *Epiphyas postvittana* (Walker), can avoid plant tissue or diet expressing *Bt* toxins [36,48,49]. The choice experiment in this study failed to provide evidence that first instar *S. albicosta* are able to detect or avoid *Bt*-expressing maize tissue. These results are similar to those for first instar *S. frugiperda*, which did not avoid Cry1F *Bt* toxin in choice studies [26,48]. 

Both the on-plant neonate dispersal and silking experiments suggest that *S. albicosta* larvae are more likely to abandon plants that express *Bt* toxins (both Vip3A and Cry1F) compared to non-*Bt* plants. These results agree with several previous publications showing that other crop pests are more likely to move off-plants that express *Bt* toxins [34,50,51,52,53]. Although our study was not designed to identify if larvae would move directionally more often from a transgenic plant to a non-transgenic plant, several studies have shown that other noctuid pests, when in the presence of *Bt* and non-*Bt* plants, present asymmetrical movement from *Bt* to non-*Bt* plants [35,44,52,53]. Larvae that move from a *Bt* plant to a non-*Bt* plant may be able to recover from sub-lethal *Bt* toxin exposure. This behavior may hasten resistance development by allowing more heterozygotes to survive [34,54,55]. In an integrated refuge scenario where *Bt* and non-*Bt* plants are located in close proximity, the potential for movement between transgenic and non-transgenic plants is of even greater concern for resistance management.

Behavior in response to exposure to *Bt*-containing diet or plant tissues can also be dependent upon an insect’s susceptivity to a given *Bt* protein. For example, *Bt*-resistant *O. nubilalis* larvae were less likely to detect and avoid toxins than susceptible larvae [56]. *Striacosta albicosta* has developed resistance to the Cry1F *Bt* toxin across its historic and expanded range [6,9], including in the population used for this study. Our results show that, when exposed to Cry1F, *S. albicosta* presents similar behavior exhibited by Cry1F-resistant *O. nubilalis* populations. Choice test studies with *Bt* toxins have shown resistant larvae are more likely to be found on non-*Bt* diet [57,58]. The lack of preference among *Bt* and non-*Bt* diet have been previously described in other Lepidoptera species [26,56], and the absence of avoidance behavior to *Bt* toxins might be explained by the ability of the larvae to overcome the toxin and by the possible absence of fitness cost to Cry1F associated with resistance [26,59]. Prasifka et al. [56] has shown that *Bt*-resistant *O. nubilalis* larvae move a shorter distance with less wandering compared to susceptible larvae. Currently, we do not have access to a Cry1F-susceptible *S. albicosta* population to make comparisons; however, our results are similar to those observed in resistant *O. nubilalis* populations [56]. In addition to understanding how resistant and susceptible insects may respond differently to *Bt*-expressing plants, an understanding of how their heterozygous offspring would have important implications for resistance management. 

In contrast to the whole plant studies (on-plant neonate dispersal and silking experiments), the artificial arena studies (maize tissue choice and video tracking experiments) did not indicate that *S. albicosta* early instars were able to detect or avoid *Bt* toxins in maize tissues. Video tracking results also contradict the on-plant larval movement experiment that showed that larvae are more likely to abandon a *Bt* plant and increase wandering when exposed to *Bt* plants. Prasifka et al. [44] noticed a similar discrepancy between on-plant and Petri dish trials, with one explanation for these results being the difference in length of exposure to *Bt* toxins. In our study, neonates were exposed for 24 h to *Bt*-expressing plants during the on-plant neonate movement experiment and were pre-exposed to *Bt* maize tissues for 24 h prior to the silking experiment. In contrast, Petri dish experiments were of shorter duration (9 h for maize tissue choice experiments and 15 min for video tracking experiments). Additionally, abiotic conditions, such as light, humidity, and microclimate in artificial Petri dish arenas, are likely to vary significantly from those experienced on whole plants during the on-plant neonate dispersal and silking studies. These discrepancies highlight the importance of conducting behavioral studies under field-realistic environmental conditions and providing periods of exposure to *Bt*-expressing plant tissues for extended periods of time. Without these conditions, the result that *S. albicosta* neonates are more likely to abandon *Bt* plants than non-*Bt* plants may have been overlooked. 

These studies provide information on the behavioral response of early larval stages of *S. albicosta* when exposed to *Bt* toxins. The current work helps us to understand the complexity of neonate dispersal behavior and its important role in host selection, particularly in a seed blend refuge scenario where *Bt* and non-*Bt* maize plants will be adjacent to one another. This is the first time *S. albicosta* dispersal and silking behavior has been tested on *Bt* and non-*Bt* plants, and the results presented in this experiment are important when considering where and how *Bt* plants should be arranged in a maize field. Additional studies should consider the movement of larvae at different ages, as different stadia are expected to differ in their susceptibility to *Bt* toxins, and additional research is needed on movement under field scenarios, since artificial arena behavior experiments can be difficult to extrapolate to field behavior [26,56]. 

## Figures and Tables

**Table 1:** Larval movement parameters (presented as mean ± SEM) measured with EthoVision video tracking software. For the variables tested non-parametrically (time in and out of the food zone), values are the mean and SEM estimates on the raw data. First instar *Striacosta albicosta*, larvae were presented with scenarios containing combinations of Vip3A, Cry1F, and non-*Bt* maize pollen in Petri dishes and recorded for 15 min. Values with different letters indicate significant differences between scenarios (*p* < 0.05). Where letters are not written, there were no significant differences.

	Pollen Choice Scenarios		
Response Parameter	Vip3A vs. Non-*Bt*	Cry1F vs. Non-*Bt*	Vip3A vs. Cry1F
Mean velocity (mm/s)	0.4175 ± 0.0370	0.5238 ± 0.0337	0.4849 ± 0.0345
Percentage of time moving	18.0203 ± 3.4050 B	32.9598 ± 3.0692 A	32.6337 ± 3.1699 A
Distance moved (cm)	36.3063 ± 3.5268	45.2766 ± 3.1790	40.8521 ± 3.2833
Time out of the food zone (s)	82.32 ± 4.48	74.33 ± 5.68	85.90 ± 4.19
Time in food zone (s)	Vip3A 7.66 ± 3.62	Cry1F 16.07 ± 5.24	Vip3A 5.64 ± 2.02
	Non-*Bt* 10.12 ± 3.43	Non-*Bt* 9.60 ± 3.71	Cry1F 8.46 ± 4.00

## Data Availability

Complete data are available upon request to the corresponding author.
